# The regulatory state of nonalcoholic steatohepatitis and metabolism

**DOI:** 10.1002/edm2.113

**Published:** 2020-10-21

**Authors:** Stephanie O. Omokaro, Julie K. Golden

**Affiliations:** ^1^ Division of Gastroenterology and Inborn Errors Products (DGIEP) OND CDER U.S. Food and Drug Administration Silver Spring MD USA; ^2^ Division of Metabolism and Endocrinology Products (DMEP) OND CDER U.S. Food and Drug Administration Silver Spring MD USA

## BACKGROUND

With nearly 100 million people affected in the United States with nonalcoholic fatty liver disease (NAFLD) and projections for the advanced stages of NAFLD to soon become the leading indication for liver transplantation, nonalcoholic steatohepatitis (NASH) remains a significant area of unmet medical need. Therapeutic options for NASH are a critical priority for clinicians, drug developers and regulatory authorities. The epidemic proportions have led to a surge in the development of novel drugs aimed at the complex pathogenesis of NASH and in regulatory submissions. There are currently no approved drugs in the United States for the treatment of adult or paediatric NASH. Potential therapeutic targets include steatosis, glucose metabolism, lipogenesis, oxidative stress, apoptosis, fibrosis and immunomodulation, all intended to alter the pathophysiology of NASH and remedy its associated complications.

While over 50 candidate drugs are currently under development in the United States, it has yet to be determined the extent to which single agents will be impactful on the multifactorial aetiology of NASH based on publicly available preliminary data.^1^ The primary challenges in NASH drug development have included the appropriateness of biopsy‐based end‐points and related statistical handling, drug development for cirrhosis, a cure‐based focus (ie only a handful of programmes evaluating symptom‐based indications such as ascites, hepatic encephalopathy and variceal bleeding) and paediatric drug development. In addition to the possible role for combination therapies, novel approaches are likely needed within discovery and regulatory science to actualize health solutions for NASH patients across the globe. Such solutions should include further exploration of the relationship between NASH and other metabolic diseases of energy homeostasis such as obesity, dyslipidaemia and type 2 diabetes mellitus (T2DM). This article will discuss the current regulatory landscape for NASH and examines the overlap of the regulatory perspectives between NASH and other metabolic diseases.

## FDA APPROVAL PATHWAYS

The FDA currently supports commercial drug development in NASH with advanced stage fibrosis as these patients are at a higher risk for liver‐related adverse clinical outcomes. There are two types of regulatory approval pathways: traditional (also known as regular or “full”) and accelerated approval. Traditional approval relies on clinical benefit end‐points which directly measure how a patient feels, functions and survives (eg morbidity and mortality) and would require durations in the order of decades for trials in precirrhotic NASH patients.

Accelerated approval^2^ is one of FDA's expedited programmes intended to facilitate drug development for serious medical conditions, for example those with unmet need, lack available therapy or where lengthy trials would be required to measure the direct clinical benefit of a drug. This pathway ensures that therapies for serious conditions are available to patients as soon as it can be concluded that the therapies' benefits justify their risks. Accelerated approval relies on appropriate surrogate or intermediate clinical end‐points that are more readily measured and considered reasonably likely to predict clinical benefit. A post‐market study to further define the clinical benefit and confirm the prediction is generally underway at the time of accelerated approval. Due to the long duration needed to assess NASH outcomes, an accelerated approval pathway using biopsy‐based surrogate end‐points that are reasonably likely to predict clinical benefit in noncirrhotic NASH has been recommended as discussed below.

## FDA DRAFT NONCIRRHOTIC NASH WITH LIVER FIBROSIS GUIDANCE—EFFICACY AND SAFETY CONSIDERATIONS

FDA draft guidance regarding drug development in noncirrhotic NASH with liver fibrosis was published in 2018^3^ with primary aims to facilitate clinical development of drugs for the treatment of noncirrhotic NASH patients with liver fibrosis who are at risk for liver‐related adverse outcomes.

The target population for these trials should have a histological diagnosis of NASH with liver fibrosis based on a NAFLD activity score (NAS) equal to or >4 with at least 1 point each in inflammation and ballooning in addition to a NASH Clinical Research Network fibrosis score of stage 2 or 3 prior to enrolment. Currently, there is no specific weight criterion for enrolment, but weight should be stable for at least 3 months prior to enrolment. Patients with T2DM can also be enrolled in NASH clinical trials if at least moderately controlled and on stable doses of anti‐diabetic medications for at least 3 months. Concomitant medications with the potential to confound the interpretation of efficacy or safety, for example by contributing to the therapeutic effect of a drug for NASH (eg Vitamin E or pioglitazone), should either be discontinued or have been on a stable dose for at least 6‐12 months.

Biopsy‐based end‐points of (a) resolution of steatohepatitis on overall histopathologic interpretation and no worsening of fibrosis, (b) one or more stage reduction of fibrosis with no worsening of NAS or (c) both improvement of NAS and fibrosis are the primary basis to support accelerated approval. A candidate drug's effect(s) on the end‐point(s) will be dependent on the mechanism of action of the drug with a potential for improvement in either NAS or fibrosis, both or neither in an individual patient. Phase 4 (post‐market) confirmatory trials verifying the clinical benefit of these histological end‐points and using composite outcome end‐points of progression of cirrhosis, reduction of decompensation events, changes in model for end‐stage liver disease (MELD) score, liver transplant and all‐cause mortality, should generally be underway at the time of submission of the marketing application for accelerated approval. End‐point selection and planned statistical testing should be discussed with the FDA review division on a case by case basis.

The guidance was posted for public comments in December 2018.^3^, ^4^ FDA considers these comments prior to finalizing the guidance in order to make revisions where appropriate.

Presently, there are 734 global NASH studies in various stages of completion, of which 298 are being conducted in the United States.^1^ Submitted protocol sample sizes have ranged from 300 to 500 subjects in phase 2 trials, 1000‐2000 subjects in pivotal phase 3 trials and 1000‐2500 subjects in phase 4 trials. Histology remains an imperfect reference standard for NASH diagnosis and monitoring because its use is accompanied by drawbacks including the invasiveness of liver biopsies (particularly in paediatric patients), sampling error due to at most 1/50 000th of the entire liver sampled, intra‐ and interobserver variability, costs and a low but observable risk of serious complications. While it is not yet known whether the effect of any particular drug will be precisely and comprehensively characterized by the inflammatory and fibrotic components of NASH injury as assessed by the histological method or whether the current 12‐18 months duration of clinical trials will be sufficiently long to allow detection of treatment effects, what is known is that the fibrosis component of the NASH pattern of injury is the strongest predictor for adverse outcomes in patients.^5^ The early failed trials in NASH,^6^ while instructive in identifying certain trial issues such as the placebo effect and the natural course of disease, provided limited information in elucidating the causes of trial failure. For example, it is unclear whether the expected changes in histology were too subtle for current modalities of detection such as the one‐stage reduction in fibrosis, and/or whether a much longer period of time is required prior to assessments when considered in the context of a life‐long chronic disease. In terms of the latter, there could be ethical concerns raised if patients were asked to enrol in exceedingly long outcome trials without preliminary evidence of benefit during the conduct of such trials. However, there is little dispute that knowledge obtained from trials with negative findings in conjunction with the increased collaborative efforts in collection of natural history information and biomarker development^7^, ^8^, ^9^, ^10^ are laying the foundation for future NASH trial designs.

The described limitations and complications of biopsy assessments support the urgent need of noninvasive diagnostic tools through increasing research and fit‐for‐purpose use. Currently, multiple biomarkers^11^ for NASH including biochemical, imaging, genetic and various omics platforms are being explored in early phase clinical trials. Some of the important regulatory considerations for biomarker use in drug development are the use of standardized definitions,^12^ identification of appropriate context of use(s), qualification^13^ for specified drug development needs and validation through multiple studies. The feasibility of optional/voluntary biopsies performed concurrently with noninvasive tests, and outcome assessments should be considered in NASH clinical trials for obtaining a potential correlation of results between these modalities.

DGIEP has employed the recommendation of a much smaller alpha (ie probability of concluding that there is a treatment effect, when in fact there is none) to achieve statistical significance for biopsy‐based NASH end‐point(s) in phase 3 trials and allow greater certainty in predicting the relationship between histology and clinical benefit. Another increasingly recognized challenge that is essential to trial design is maintenance of ongoing trial conduct and integrity after accelerated approval or public dissemination of interim data which should be prospectively planned. A detailed unblinding plan, careful selection of database lock and cut‐off date, projections of patient adherence and retention, subject awareness through updates to the informed consent document, and measurement of operational characteristics over time are important considerations for maintaining post‐approval, ongoing trial integrity.

For clinical outcome trials, there may be drivers of the clinical benefit end‐point (eg progression to cirrhosis component may contribute a greater number of events as it will occur more frequently than other components comprising the composite end‐point); while this may allow earlier completion of trials in a shorter duration, it may not evaluate all aspects of the disease and this will need to be factored into the data review and analysis to ensure that a positive treatment response is not an isolated effect.

## FDA DRAFT NASH WITH COMPENSATED CIRRHOSIS GUIDANCE—EFFICACY AND SAFETY CONSIDERATIONS

FDA draft guidance regarding drug development in NASH with compensated cirrhosis was published in 2019.^14^ Drug development for NASH with cirrhosis is challenged with appropriately defining this inherently higher risk population for clinical trials and recognizing end‐points that have an observable impact on the advanced disease physiology. Noninvasive evidence of cirrhosis may be acceptable in early phase trials, while trials intended to support a marketing application should provide evidence of histologic confirmation of the treatment effect. The current emphasis is placed on trials in a compensated cirrhosis population with end‐points of reduction of decompensation events; however, composite end‐points using markers of liver synthetic and functional capacity could be explored in current and future trials to counter the potential for heterogeneity in the target population secondary to the substages of cirrhosis. In general, FDA has not recommended combining precirrhotic and cirrhotic patients in the same trial because of the differences in monitoring and management which will likely complicate trial design. If both patient populations are included in the same trial, separate inclusion/exclusion criteria, independent powering of the two subpopulations and a differential schedule of clinic monitoring are recommended. Significant efficacy results in the overall trial population will need to be supported by positive results from each of the subpopulations. The guidance does not recommend inclusion of patients with decompensated cirrhosis or those nearing a decompensated stage because the clinical status of these patients may not be sufficiently stable for the recommended duration of these trials.

At present, FDA recommends that clinical trial protocols in populations with NASH with compensated cirrhosis not exceed 25%‐30% cryptogenic cirrhosis patients, a proportion similar to that expected for the upper range^15^ of real‐world biopsies, unless otherwise adequately justified. Trials agnostic to cirrhosis aetiology may avoid the challenge of attributing NASH causality in the diagnostic dilemma of cryptogenic cirrhosis.^16^ The Agency has encouraged obtaining historical biopsies to confirm prior presence of steatohepatitis to support a diagnosis of NASH as the underlying aetiology when enrolling patients with cryptogenic cirrhosis. Trials examining symptomatic improvement of cirrhosis complications such as ascites, variceal bleeding and hepatic encephalopathy currently represent only a small proportion of the NASH‐trial landscape but are greatly needed in improving morbidity and symptomatic burden of patients while awaiting development and approval of disease‐modifying therapeutic options. The perceived challenges of such trials may be related to the disease severity of the target population, lack of available validated patient‐reported outcome (PRO^17^, ^18^, ^19^) instruments, associated regulatory requirements and few examples of approved labelling based on PROs within FDA in general. Use of fit‐for‐purpose instruments with appropriate conceptual frameworks and evidence of content validity along with early engagement with the FDA are necessary to promote drug development that incorporates the patient voice.^20^

The primary safety conundrum for NASH drug development is that investigational agents intended to treat liver disease can also result in liver injury.^21^ For this reason, early hepatic impairment studies are encouraged to better characterize the study drug prior to studies in higher risk populations (eg cirrhosis) and because patients may progress to cirrhosis during the conduct of clinical trials. Detailed drug‐induced liver injury (DILI) evaluation and management algorithms in addition to close monitoring as per the DILI guidance^22^ are recommended in the setting of suspected DILI. While the DILI guidance may not be fully applicable to patients with underlying liver disease and elevated liver biochemical baselines, it remains the cornerstone for liver safety monitoring in clinical trials. The Agency is aware of this knowledge gap and is engaged globally in collaborative discussions^23^, ^24^ to address the need for guidelines specific to patients with pre‐existing liver disease.

## NASH IN THE REGULATORY CONTEXT OF OBESITY AND OTHER METABOLIC DISEASES

It has been estimated that as many as 75% of patients with obesity have NAFLD and 20% have NASH.^25^, ^26^ Obesity, insulin resistance, T2DM and dyslipidaemia increase the risk of progression from nonalcoholic fatty liver (NAFL) to NASH.^27^, ^28^ The treatment of obesity provides an opportunity to simultaneously address co‐existing metabolic diseases, including NASH. Likewise, weight loss strategies—including lifestyle and surgical interventions—have the potential to improve outcomes in NASH patients by normalizing liver enzymes, inducing regression in hepatic pathology, and mitigating cardiovascular risk factors.^5^, ^29^ There are limited data to suggest whether drug‐induced weight loss can directly mediate these effects; however, therapies that could effectively treat obesity, its related co‐morbidities and NASH are highly desirable.

The most recently updated FDA draft guidance for weight management was published in 2007.^28^ The draft guidance outlines the patient population, programme size and duration, and end‐points for evaluation of obesity drugs in all phases of development. Patients with BMIs greater than or equal to 30 kg/m^2^ or greater than or equal to 27 kg/m^2^ in the presence of weight‐related co‐morbidities are thought to be at significant risk for weight‐related morbidity and mortality that would justify the use of drug treatment. The draft guidance cites the examples of T2DM, hypertension, dyslipidaemia, sleep apnoea and cardiovascular disease as weight‐related co‐morbidities. Although not specifically discussed in the draft guidance, obesity drug trials likely enrol many patients with NAFLD given the overlap in these populations. Special attention should be given to enrolment criteria and safety monitoring plans when including a significant proportion of NASH patients, particularly in those drug development programmes that have demonstrated potential for nonclinical and/or clinical signals of liver toxicity.

Weight change from baseline is the primary efficacy end‐point in placebo‐controlled trials for obesity drugs, and the guidance specifies that weight loss should be demonstrated over the course of at least one‐year duration. The goals of weight loss in obesity management are to prevent or slow the progression of obesity‐related health outcomes and improve quality of life. Weight loss of 5 per cent—evaluated as mean change from baseline and in a categorical analysis of the proportion of patients losing 5 per cent body weight—is generally considered clinically meaningful in patients with obesity as it has been associated with improvements in cardiometabolic biomarkers, such as blood pressure, lipids and fasting glucose.^30^

There are currently 5 drugs that are FDA‐approved for chronic weight management in patients with obesity: orlistat (gastrointestinal lipase inhibitor), lorcaserin (serotonin 2C receptor agonist), phentermine/topiramate (combination of a sympathomimetic anorectic and an antiepileptic drug), bupropion/naltrexone (combination of an aminoketone antidepressant and an opioid antagonist) and liraglutide (GLP‐1 receptor agonist). One‐year placebo‐subtracted weight loss from baseline body weight as described in the prescribing information ranges from 3% to 9% and depends to a large extent on the patient population, background lifestyle intervention, treatment adherence, study discontinuation rate and the statistical methods used to address missing data. The labelled prescribing information for these drugs includes changes in weight‐related secondary end‐points, such as blood pressure and lipids, but currently does not include liver‐related efficacy end‐points, or claims related to the reduction of cardiovascular morbidity and mortality or improvement in quality of life. To date, no obesity drug has demonstrated cardiovascular risk reduction in a dedicated trial.

## PAEDIATRIC CONSIDERATIONS

Obesity and severe obesity in children and adolescents ages 2 and above are often defined as a BMI at or above the 95th percentile of sex‐specific BMI‐for‐age and BMI at or above 120% of the 95th percentile, respectively.^31^ With the rise in paediatric obesity over the last several decades, the prevalence of associated metabolic diseases including NAFL and NASH is also increasing.^32^, ^33^, ^34^ As with adults with obesity or NASH, the mainstay of treatment of children and adolescents is lifestyle modification, with the hope and expectation that effective diet and physical activity interventions can prevent or delay many of the associated co‐morbidities.

Currently, there are limited pharmacological treatment options in children and adolescents with obesity, with only one drug, orlistat, labelled for long‐term weight management in patients ages 12 and above. The assessment of certain drugs and biologics in children is required under the Pediatric Research Equity Act,^35^ and new drugs to treat obesity in children are currently under evaluation. Primary end‐points in studies to evaluate obesity treatment in growing children are typically based on changes in BMI or related parameters, and the selection of primary and secondary end‐points depends upon the patient population, research question and drug. This is an active area of research, and sponsors are encouraged to discuss their paediatric plans for obesity drugs with the Agency.

Paediatric studies in NASH are few and, as with adults, there are no FDA‐approved drugs. While several drug manufacturers have identified the need for paediatric studies in NASH as early as possible in their initial paediatric study plans, the path forward is less clear because of the differences in adult and paediatric NASH histopathologic features making it impracticable to extrapolate efficacy from adult data to children, lack of natural history information to define feasible end‐points, ethical and regulatory requirements that enrolment of children in drug intervention trials be adequately justified through demonstration of prospect of direct benefit^36^ to the subject and the need for age‐appropriate formulations. Notably, the pressing need for end‐points with acceptable invasiveness that can be assessed in a timely manner and will predict clinical outcomes cannot be overstated. There has been some suggestion that assessing delays in the time to onset or prevention of NASH co‐morbidities may be possible end‐points; however, the sample size and trial duration needed for such paediatric studies as well as the clinical relevance or likelihood that these would predict direct clinical benefit for NASH may still be limiting factors.

## AREAS OF EXPLORATORY OPPORTUNITIES

Historically, drugs used to treat obesity have focused on the primary end‐point of weight and amelioration of traditional weight‐related co‐morbidities, such as T2DM and dyslipidaemia. However, given the overlap of these conditions with NAFLD, drugs to treat obesity are being considered for treatment of NASH.^37^

Drug development and regulatory pathways in metabolic and endocrine disorders are increasingly intersecting with NASH through overlapping patient populations, drug classes and mechanisms of action, end‐points and clinical trial designs. At least 25% of the active NASH Investigational New Drug (IND) submissions within FDA have benefited from collaboration between DGIEP and DMEP. Similarly, programmes regulated within DMEP for metabolic diseases such as obesity and dyslipidaemia have explored liver‐related end‐points and enrichment with NASH populations. For these reasons, patients with NASH could be specifically targeted in obesity and other metabolic drug programmes to enrich for cardiometabolic risk factors, or to evaluate NASH end‐points as part of the efficacy assessments to support obesity drug approval. However, equating benefit from one metabolic disorder to the next may be more complicated than foreseen even if it were possible to attribute relatedness of differential outcomes (ie improvement of metabolic derangements equals an improvement in NASH). As this is a fairly novel approach in already complex pathophysiology, drug manufacturers and investigators are encouraged to discuss such study proposals with the Agency.

Examples of overlapping drug classes or mechanisms in the pipeline for NASH and other metabolic diseases include glucagon‐like peptide‐1 receptor agonists, peroxisome proliferator‐activated receptor agonists, sodium glucose cotransporter‐2 inhibitors, fibroblast growth factor‐21 analogs, and others.^1^ Complementary targets and mechanisms create opportunities for approved drugs and investigational agents for other metabolic indications to be reconsidered or used as part of combination therapies, to potentially expand the armamentarium for NASH. Combination drug development programmes will need to address the fixed combination rule, which states that “[t]wo or more drugs may be combined in a single dosage form when each component makes a contribution to the claimed effects and the dosage of each component (amount, frequency, duration) is such that the combination is safe and effective for a significant patient population requiring such concurrent therapy as defined in the labelling for the drug”.^38^ A draft rule^39^ was published on 23 December 2015 that proposes to revise the regulations governing fixed combination drug products. Although the proposed rule has not yet been finalized, the preamble to the proposed rule describes FDA's longstanding policy on how to demonstrate the contribution of each component of the fixed combination drug to the claimed effects.

A fresh look at the Venn diagram of the pathophysiology for these metabolic diseases may be needed to develop emerging treatments aimed at common pathways in addition to downstream effects. The convergence of risk factors, patterns of injury, drug mechanisms of action, biomarkers, outcomes and regulatory frameworks may prove insightful in generating the next wave of therapeutic options. The future remains exciting and promising for NASH drug development.

## CONFLICT OF INTEREST

The authors declared no conflict of interest.

## AUTHOR CONTRIBUTIONS

SOO conceived the concept for the manuscript and is the primary author. JKG authored sections on drugs to treat obesity and other metabolic diseases. Both authors contributed to the preparation and revision of the manuscript.

## ETHICAL STATEMENT

The authors declare that the manuscript is original and has not been submitted for publication elsewhere. No specific ethical approval or informed consent was required for this editorial.
